# Tuberculosis arthritis of the metatarsal phalangeal: a rare location

**DOI:** 10.11604/pamj.2014.17.323.4220

**Published:** 2014-04-29

**Authors:** Mohamed Ali Berrady, Ismael Hmouri, Amine Benabdesslam, Mohamed Salah Berrada, Moradh El Yaacoubi

**Affiliations:** 1 Department of Orthopaedic Surgery and Traumatology, University Hospital Center Ibn Sina, Rabat, Morocco

**Keywords:** Tuberculosis, osteoarthritis, metatarsophalangeal joint, anti tuberculous chemotherapy, sequelae

## Abstract

Tuberculosis TB is common in countries constituting endemic areas like Morocco, spinal sites represents half of osteo-articular locations, while peripheral locations in the limbs are rare. The authors relate in this observation the case of a particular location of tuberculosis arthritis. It is osteoarthritis of the metatarsophalangeal joint of the 2^nd^ ray of the foot. Clinical signs were characterized by a moderately painful swelling of the dorsum of the foot with slow evolution. The definitive diagnosis was histologically obtained. Clinical cure was achieved after 09 months of medical treatment.

## Introduction

Osteo articular tuberculosis represents 2 to 5% of all locations [1, 2], spinal localization represents 50% of bone lesions [3–7], and the rest in decreasing order: pelvis, hip and femur, knee and tibia, ankle or shoulder, elbow or wrist, multifocal affections. We report in this observation the case of a patient with tuberculous osteoarthritis of the metatarsophalangeal joint of the second ray of the left foot, observed and treated in the orthopedic department and trauma surgery CHU IBN SINA in Rabat, MOROCCO, with an epidemiological, clinical and therapeutic reminder of this disease.

## Patient and observation

**Figure 1:**
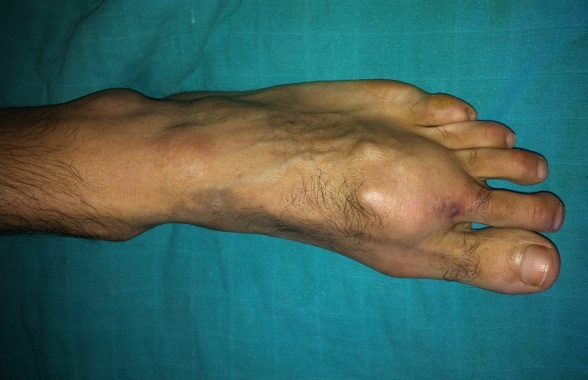
Clinical appearance before treatment

**Figure 2:**
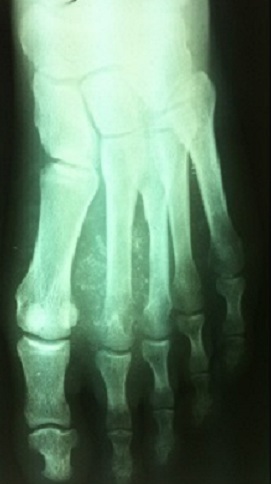
Clinical appearance before treatment

**Figure 3:**
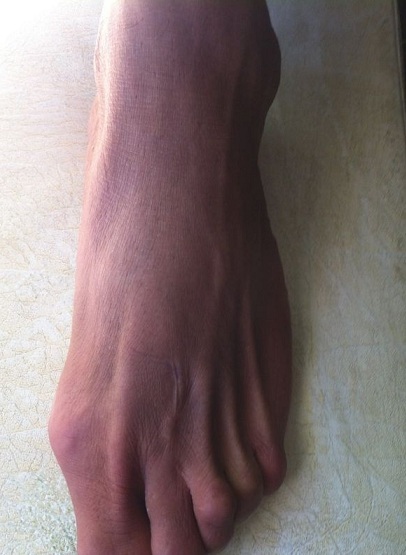
After 09 months of medical treatment

**Figure 4:**
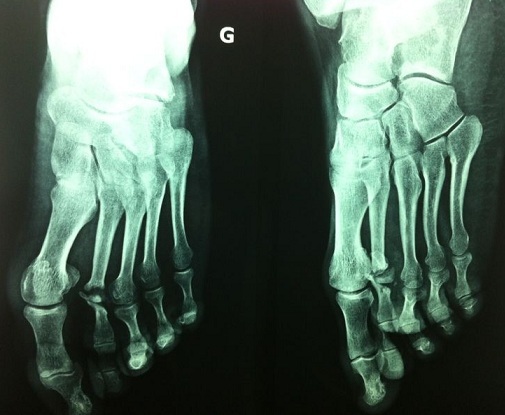
After 09 months of medical treatment

It is a patient of 53 years with no individual's pathological history including no prior tuberculosis or TB concept of contagion. The patient is from disadvantaged socio-economic backgrounds, who consult for swelling of the dorsum of the foot, lasting for more than a year, slightly painful, without systemic signs or weight loss. His vaccination status was not accurate. The patient had previously consulted with a general practitioner and had been under antibiotics and analgesics without improvement. Clinical examination revealed an indurated swelling, delicate, fixed relatively to the two planes with ipsilateral inguinal lymphadenopathy (Figure 1). Laboratory tests revealed an inflammatory anemia to 10.2 g/dl with a slightly accelerated sedimentation rate (25mm). radiographs of the foot revealed a near-total destruction of the metatarsophalangeal joint of the 2nd toe with densification of the adjacent soft tissues with images of calcifications (Figure 2). A surgical biopsy was performed. Its result confirmed the diagnosis of tuberculous osteo arthritis by highlighting a giganto - epithelioid cell granuloma with presence of caseum. The patient was placed in a TB protocol for a period of 9 months according to the outline 2RHZE/7RHE. The monthly checks revealed clinical improvement with a reduction in the volume of the swelling and pain relief without side effects related to anti-TB. Radiological control showed a marked regression of bone destruction with sequelae images localized to the neck of the second metatarsal and the base of the proximal phalanx (Figure 3), (Figure 4). The patient seemed to be very satisfied with the result and was lost from sight.

## Discussion

Articular bone tuberculosis represents 2-5% of all locations; the vertebral involvement is predominant with 50% of cases, limbs affections meanwhile, remain relatively rare. Coexistence of TOA and visceral tuberculosis or lymph node is present in 40 -20% of cases [2, 3, 8, 9]. We distinguish on the one hand arthritis and osteo arthritis, and on the other hand osteitis and osteomyelitis (less frequent).

Epidemiologically, TOA classically affected children more than adults [10], but now the proportion of adults and children are affected very little different. There is probably no sex predominance [8, 9, 11]. It appears that the TOA is usually unifocal, multifocal forms vary between 3 and 20% of cases in the series [4, 2, 7, 10, 12]. TOA is a pauci bacillary form in which the MYCOBACTERIUM TUBERCULOSIS is quiescent type or slow multiplication. It is often accompanied by one or more pus-filled abscesses or caseum.

The clinical manifestations of TOA realize a subacute or chronic arthritis table, progressing to progressive worsening over several weeks or months as seen in the case of our patient. Time between first symptoms and diagnosis of sureness varies depending on the series; it was between 13.6 and 21 months in two large series [4, 7], 14 months in our observation.

Pain, joint swelling and lameness are the usual signs. Pain may be mechanical, inflammatory or mixed. Joint swelling may be due to effusion (55% of cases) [4, 7], an abscess (20A 25%) [4, 7] or to synovial hypertrophy. The presence of a fistula can be reported and examination of lymph node areas should be carried out in search of a lymphadenopathy, in the case of our patient an inguinal lymphadenopathy occurred one month after the onset of pain. General signs and more specifically signs of tubercular infiltration are suggestive but unfortunately inconstant (20-45% of cases) [1, 4, 7, 8, 13].

While many authors relate the notion of trauma as a trigger or promoting factor in the development of TOA, we could say beyond doubt that it plays a role in the reactivation of dormant KOCH bacillus; it could be simply the reason for consultation. OAT or arthritis of the foot and ankle often start with osteitis with secondary articular extension. Literature reports two series localized to the foot, MARTINI with 69 cases and the DHILLON with 74 cases, with 26% of midfoot and the Lisfranc joint affection. All joints of the foot may be affected and all associations are possible, however the location in the midfoot and the LISFRANC seem to be predominant. Diagnosis is often delayed as in our case and functional sequelae are frequent. The blood tests are not very specific. They objectify in addition to an inflammatory anemia, an increased erythrocyte sedimentation rate between 25 and 100 mm the first hour in the majority of cases. Analysis of joint fluid in case of ponctionable effusion shows the presence of an inflammatory type liquid with presence of 5,000 to 50,000 elements per mm cube.

Moroccan study describing the analysis of 30 tuberculous arthritis puncture liquid, the average cellularity was 14,520 per cubic mm, with a predominance of neutrophils in 60 to 80%, and lymphocytes in 20 to 30% of cases. The tuberculin skin test is positive in 80-85% of cases but remains very limited especially in endemic contexts such ours. More recently, researchers have tried to develop more specific and faster methods especially for the diagnosis of TB; it is the case of the PCR (polymerase chain reaction). Many authors report a specificity of about 92% of 98 of the PCR; therefore it may be particularly interesting in the TOA.

Two associations are possible but exceptional; the first is the association between gout and arthritis tuberculosis with concomitant presence of urate crystals and BK in culture [14]. The second, more rare, is chondrocalcinosique tuberculous arthritis association. Radiologically, OAT performs the classic triad of Phemister gathering juxta articular osteoporosis, bone erosions and progressive narrowing of the joint space. It remains true that the radiological signs depend intimately on the stage which the diagnosis has been suspected, indeed radiographs at an early stage may be normal [15] or show at most hyper transparency of epiphyses and densification with increased volume of soft tissues ( 7to 36%) by series [5, 4, 7]. Then appears the osteolysis with geodes and erosions. Martini [10] proposed a radiological classification with four stages from discrete epiphyseal osteoporosis to the complete destruction of the joint with joint deformity.

Bone scintigraphy has the advantage to search for other locations of osteo_articular tuberculosis which are clinically silent, it almost constantly objective articular uptake [5]. MRI is the best imaging test in terms of diagnostic study and monitoring of OAT, it allows studying accurately synovial hypertrophy, joint effusion, cartilage destruction etc.... Unfortunately, it remains expensive in some countries like ours; it was not performed for our patient. The definitive diagnosis is made by histological examination; it is typically giganto epithelial cell granuloma with caseous necrosis.

It is useful to remind that the diagnosis of tuberculous infection can be confirmed by the isolation of BK in other environements such as sputum, this is why it would be interesting to seek a visceral or lymph node tuberculosis before any suspicion of TOA. Indeed, in the Moroccan series BOUAZZA and cl [7], the diagnosis of tuberculosis was obtained outside the articular joint fluid analysis in 17% of cases.

Therapeutically, treatment of TOA remains essentially medical; it uses a combination of effective antibiotics individually on the BK. So far, there is no consensus on the treatment protocol or the duration of treatment o undertake. For the north - americans., the duration of treatment should be between 06 and 09 months providing that rifampicin's used [16, 17]. In francilienne series of 206 cases, the duration of treatment was 14 months for spinal tuberculosis and 13 months for extra vertebral TOA [10]. In our case, we opted for a practical protocol based on Isoniazid, Rifampicin, Pyrazinamide and ETAMBUTHOL for a period of 09 months.

Regarding surgery, whether first-line, second-line or functional target is less important. Surgical treatment, when it is undertaken, is usually to make a gesture of joint debridement, synovectomy or drainage of an abscess [6–17]. Appart from the biopsy, we did not conduct surgical treatment in our case. According to the literature the rate of healing of the TOA well treated exceeds 90% [18]. The main cause of failure of a TOA diagnosed early remains poor adherence. Diagnostic and therapeutic delay is responsible for the occurrence of functional sequelae. In case of TOA, a sequelae stiffness was noted in 68% of cases [19]. For children, due to cartilage growth damage, these effects are more substantial (shortening or angulation of the member may occur).

## Conclusion

Articular bone TB remains a public health problem in developing countries like MOROCCO. Its management requires close collaboration between orthopedic, bacteriologist and hygienist. Clinical signs are generally little suggestive and leads in most cases to a late diagnostic and late therapeutic which cause irreversible damage. Certainty diagnostic remains histological and treatment is essentially medical.

## References

[1] Evanchick CC, Davis DE, Harrington TM (1986). Tuberculosis of peripheral joints: an often missed diagnosis. J Rheumatol..

[2] Monach PA, Daily JP, Rodriguez-Herrera G, Solomon DH (2003). Tuberculous osteomyelitis presenting as shoulder pain. J Rheumatol..

[3] Watts HG, Lifeso RM (1996). Tuberculosis of bone and joints. J Bone Joint Surg Am..

[4] Pertuiset E, Beaudreuil J, Horusitzky A, Lioté F, Kemiche F, Richette P (1997). Aspects épidémiologiques de la tuberculose ostéo-articulaire de l'adulte: Étude rétrospective de 206 cas diagnostiqués en région parisienne durant la période 1980-199. Presse Méd.

[5] Houshian S, Poulsen S, Riegels-Nilesen P (2000). Bone and joint tuberculosis in Denmark. Increase due to immigration. Acta Orthop Scand..

[6] Pertuiset E, Beaudreuil J, Lioté F, Horusitzky A, Kemiche F, Richette P (1999). Spinal Tuberculosis in adults (A study of 103 cases in a developed country, 1980-1994). Medicine (Baltimore)..

[7] Benbouazza K, El Maghraoui A, Lazrak N, Bezza A, Allali F, Hassouni F (1999). Les aspects diagnostiques de la tuberculose ostéo-articulaire: Analyse d'une série de 120 cas identifiés dans un service de rhumatologie. Sem Hôp Paris..

[8] Garrido G, Gomez-Reino JJ, Fernandez-Dapica P, Palenque E, Prieto S (1988). A review of peripheral tuberculous arthritis. Semin Arthritis Rheum..

[9] Teklali Y, Fellous El Alami Z, El Madhi T, Gourinda H, Miri A (2003). La tuberculose ostéo-articulaire chez l'enfant (mal de Pott exclu): à propos de 106 cas. Rev Rhum Mal Ostéoartic..

[10] Martini M (1988). La tuberculose ostéo-articulaire.

[11] Chang JH, Kim SK, Lee WY (1999). Diagnostic issues in tuberculosis of the ribs with a review of 12 surgically proven cases. Respirology..

[12] Tuli SM (2002). General principles of osteoarticular tuberculosis. Clin Orthop Relat Res..

[13] Dhillon MS, Nagi ON (2002). Extra spinal tuberculosis: tuberculosis of the foot and ankle. Clin Orthop..

[14] Lorenzo JP, Csuka ME, Derfus BA, Gotoff RA, McCarthy GM (1997). Concurrent gout and Mycobacterium tuberculosis arthritis. J Rheumatol..

[15] Babhulkar S, Pande S (2002). Extra spinal tuberculosis: unusual manifestations of osteoarticular tuberculosis. Clin Orthop Relat Res..

[16] Allali F, Mahfoud S, Hajjaj-Hassouni N (2002). Caractéristiques du liquide articulaire dans la tuberculose ostéo-articulaire. Rev Rhum Mal Ostéoartic..

[17] Griffith JF, Kumta SM, Chung Leung P, Cheng JC, Chow LT, Metreweli C (2002). Imaging of musculoskeletal tuberculosis: a new look at an old disease. Clin Orthop Relat Res..

[18] Pertuiset E, Beaudreuil J, Horusitzky A (1999). Traitement médical de la tuberculose ostéo-articulaire: Étude rétrospective de 143 cas chez l'adulte. Rev Rhum Mal Ostéoartic..

[19] Vuyst de D, Vanhoenacker F, Gielen J, Bernaerts A, Schepper de AM (2003). Imaging features of musculoskeletal tuberculosis. Eur Radiol..

